# Factors Associated With Right Ventricular Size Recovery After Transcatheter Closure of Isolated Atrial Septal Defects

**DOI:** 10.1016/j.jacadv.2026.103304

**Published:** 2026-10-01

**Authors:** Michael N. Gritti, Sara Pollanen, Olivia Carere, Rajiv R. Chaturvedi, Brian W. McCrindle, Lee N. Benson

**Affiliations:** a Labatt Family Heart Centre, Hospital for Sick Children, Toronto, Ontario, Canada; b Faculty of Medicine, University of Toronto, Toronto, Ontario, Canada; c Division of Cardiology, Heart Institute, Children's Hospital Colorado, University of Colorado Denver, Aurora, Colorado, USA

**Keywords:** atrial septal defect, cardiac catheterization, congenital heart disease, interventional cardiology, right ventricular remodeling

**Figure:**
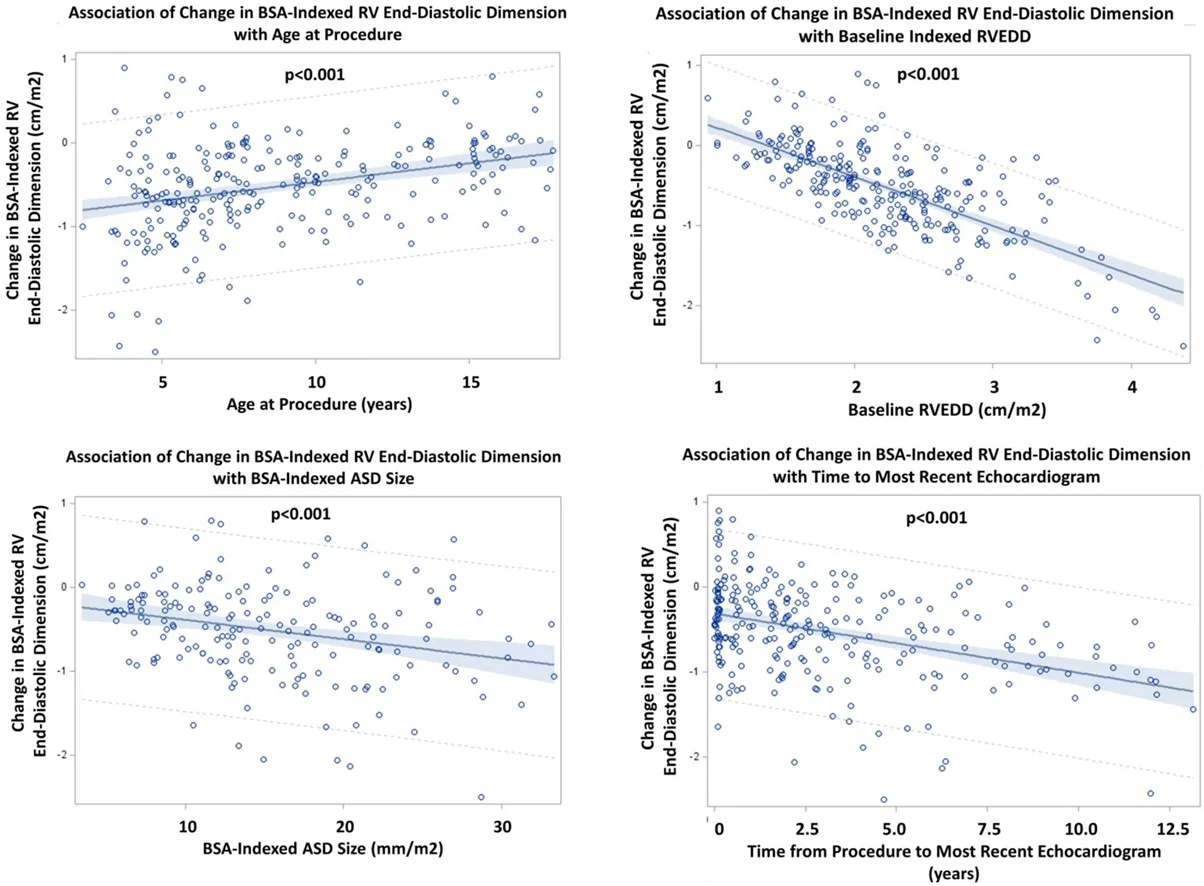

**What is the clinical question being addressed?**

This study assessed the change in right ventricular size following transcatheter closure of isolated secundum atrial septal defects in children, and what factors are associated with ventricular remodeling.

**What is the main finding?**

Transcatheter closure of atrial septal defects significantly reduces right ventricular size in children, with greater reduction linked to younger age at closure, larger defect size, greater baseline dilation, and longer follow-up.

Atrial septal defects (ASDs) account for approximately 10% of congenital heart lesions and cause progressive right heart volume overload.^1^^,^^2^ If untreated, this may lead to pulmonary hypertension, congestive heart failure, atrial arrhythmias, exercise intolerance, and premature death.^1^ Transcatheter device closure for anatomically suitable secundum ASDs has a high procedural success rate and favorable long-term outcomes in pediatric populations.^2^^,^^3^

Following closure, right ventricular (RV) remodeling is expected due to elimination of the left-to-right shunt. Although RV dimensions often improve within the first year, the extent and determinants of remodeling vary, and prior studies have largely focused on functional outcomes with limited data evaluating predictors of longitudinal RV remodeling.^4^^,^^5^ Understanding predictors of RV recovery has important clinical implications, helping to optimize timing of intervention and identifying those patients at risk of persistent RV dilation. This study evaluated changes in RV size following transcatheter closure of isolated secundum ASDs in children and identified factors associated with the degree of remodeling.

Institutional databases were searched to identify children who underwent transcatheter device closure of an isolated secundum ASD at The Hospital for Sick Children between August 1989 and November 2016. Research Ethics Board approval was obtained, and informed consent was waived due to the retrospective design. Eligible children were <18 years at the time of intervention, and had echocardiographic data available before, immediately after, and in follow-up. Children with fenestrated defects, genetic abnormalities, or concomitant congenital heart disease were excluded. ASD size was indexed to body surface area (BSA). RV size was assessed using BSA-indexed RV end-diastolic dimension (RVEDDi) at echocardiography at each time point, with corresponding Z-scores.

The primary analysis evaluated changes in RV dimensions over time, as reported by our echocardiography laboratory. Generalized linear regression analyses identified factors associated with greater reductions in indexed RV size, including age at closure, indexed ASD size, baseline RV size, and follow-up duration.

A total of 385 children met the inclusion criteria, 62% female, with a mean age of 8.9 ± 4.3 years at ASD closure. The mean weight was 31.1 ± 17.2 kg at time of closure. A residual shunt was seen in 14% (n = 54) of children. The mean BSA-indexed ASD diameter was 14.9 ± 6.6 mm/m^2^. RVEDDi decreased from 2.2 ± 0.7 to 1.7 ± 0.5 cm/m^2^ at a median follow-up of 1.8 years (range up to 13 years; *P* < 0.001). The mean RVEDD Z-score was +1.5 at follow-up (preintervention, mean RVEDD Z score +3.6), with 47% of patients maintaining a Z-score >+2. No patient had RV systolic dysfunction, or > mild tricuspid regurgitation, on preprocedure or postprocedure echocardiogram. Electrocardiographic parameters remained stable, with no significant change in QRS duration from preclosure (90 ± 12 ms) to follow-up (89 ± 12 ms; *P* = 0.35). Generalized linear regression analyses identified younger age at closure, larger indexed ASD size, greater baseline RV size, and longer follow-up as independently associated with greater RV reduction (all *P* < 0.001) (Figure 1). RV size reduction was not associated with a residual shunt.

**Figure 1:**
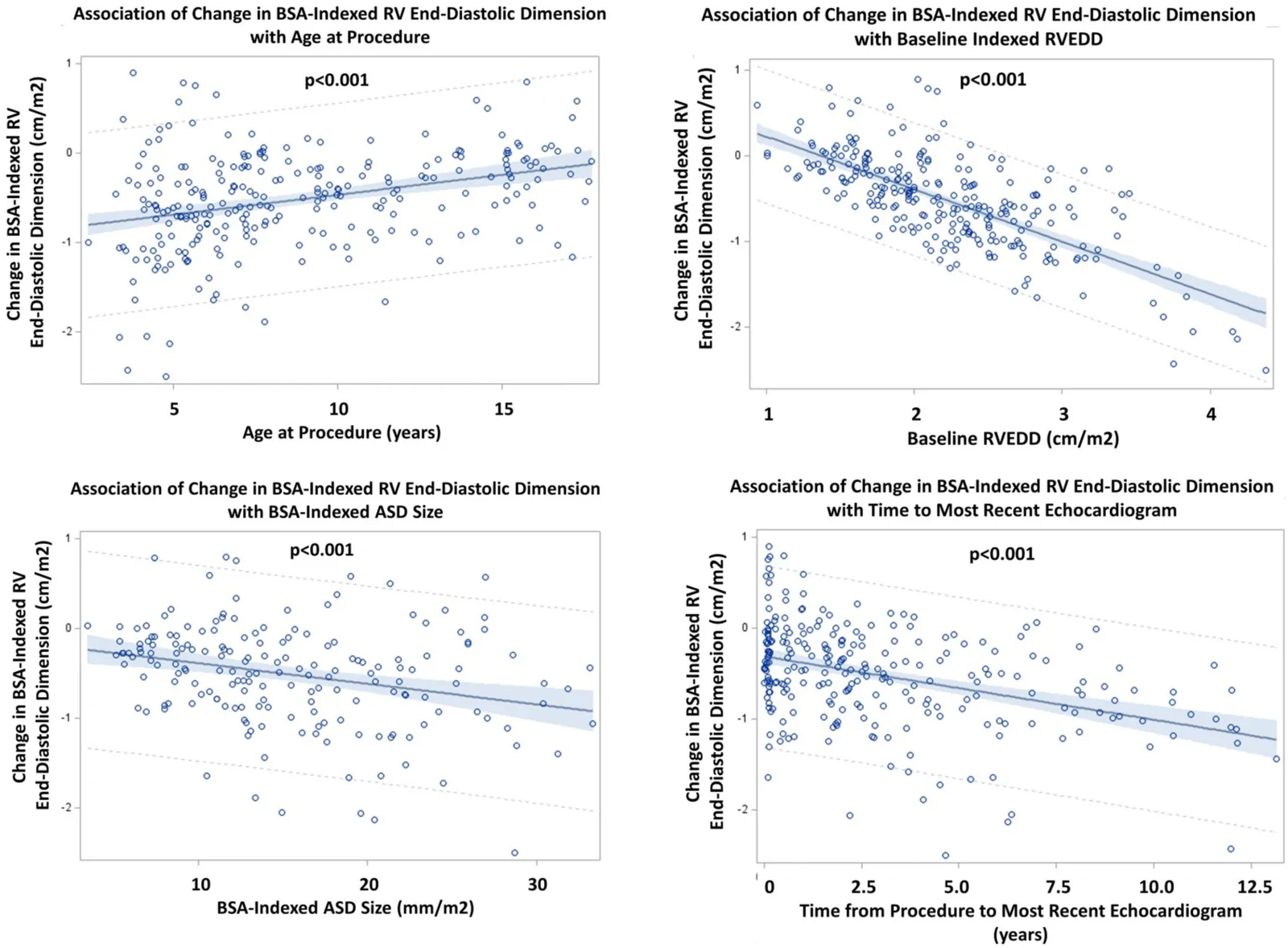
Predictors of RV Remodeling After ASD Closure Generalized linear regression analysis of factors associated with change in body surface area–indexed right ventricular end-diastolic dimension from baseline to follow-up after transcatheter closure of an isolated secundum atrial septal defect. Younger age at closure, larger indexed defect diameter, greater baseline right ventricular size, and longer follow-up were independently associated with greater reduction in right ventricular size. Shaded areas = 95% CI around the fitted regression line. Dashed lines = 95% prediction intervals for individual observations. ASD = atrial septal defect; BSA = body surface area; RV = right ventricle; RVEDD = right ventricular end-diastolic dimension.

In this cohort, transcatheter closure of an isolated secundum ASD was associated with significant reduction in RV size; however, remodeling remained incomplete at follow-up in a substantial proportion of children. Despite a marked decrease in mean RVEDDi, 47% of children continued to demonstrate RV dilation at follow-up, indicating that normalization was not complete within the first year after the procedure, warranting longer follow-up.

The association between larger defects and greater baseline RV size with more pronounced remodeling likely reflects the greater reduction in volume overload following closure. The relationship between follow-up duration and RV reduction suggests that remodeling may be a progressive process extending beyond the immediate postprocedural period.

Younger age at intervention was associated with greater improvement in RV dimensions, supporting the potential benefit of earlier ASD closure. This is consistent with prior evidence demonstrating greater structural plasticity in younger myocardium.^4^ Persistence of RV dilation may reflect insufficient follow-up to observe complete remodeling, emphasizing the importance of timely diagnosis and intervention.

The presence of a residual shunt following device closure was not associated with reduced RV remodeling, demonstrating the efficacy of contemporary transcatheter techniques. Furthermore, despite structural changes, QRS duration remained stable over time, suggesting no measurable impact of RV remodeling on ventricular conduction within the follow-up period.

This study is limited by its retrospective, single-center design and short follow-up, restricting assessment of long-term remodeling. Multicenter studies with longer follow-up are needed to define the significance of persistent RV dilation.

Transcatheter ASD closure leads to significant remodeling of the RV. Greater reductions in RV size are independently associated with younger age at closure, larger defect size, greater baseline RV dilation, and longer follow-up duration. These factors may be important to consider when planning treatment strategies aimed at optimizing structural recovery and long-term outcomes.

## Funding support and author disclosures

The authors have reported that they have no relationships relevant to the contents of this paper to disclose.
